# Prolactin Changes as a Consequence of Chemical Exposure: de Burbure and Bernard Respond

**Published:** 2006-10

**Authors:** Claire de Burbure, Alfred Bernard

**Affiliations:** School of Public Health, Catholic University of Louvain, Brussels, Belgium, E-mail: bernard@toxi.ucl.ac.be

We appreciate the letter from Alessio and Lucchini concerning the number and variety of toxicants able to affect serum prolactin levels. Reflecting on the wide variability of the currently available data, we would like to make two additional points.

The first point concerns the usefulness of serum prolactin as a potential indicator of neurotoxicity for populations at risk. This biomarker indeed appears to be influenced by a large number of both organic and inorganic chemicals, which have seemingly little in common in terms of mechanistic action (e.g., heavy metals, pesticides, styrene, polychlorinated biphenyls). Moreover, one chemical—cadmium, for example—can have a biphasic dose-dependent effect on serum prolactin (Lafuente et al. 2003), an effect we did not observe in our study (de Burbure et al. 2006) because of low exposure levels; this dose-dependent effect is reminiscent of the biphasic effects of lead on glutamate neurotransmission shown to be dependent on glycine receptor affinity (Marchioro et al. 1996).

As proposed by Alessio and Lucchini in their letter, these data reflect the complexity of the control of prolactin secretion, which is modulated not only by dopamine but also by several other neurotransmitters. These neurotransmitters include serotonin, γ-aminobutyric acid (GABA) [as demonstrated by the hyperprolactinemia developed by GABA_B1_ knock-out mice (Catalano et al. 2005)], glycine, and glutamate (Fitsanakis and Aschner, 2005; Nagy et al. 2005). In view of these neurotransmitters, serum prolactin—albeit sensitive—appears to be a rather non-specific biomarker for monitoring populations at risk; therefore, serum prolactin will likely remain a predominantly useful tool in the field of research until the multiple facets of controlling prolactin secretion are unveiled.

Another important issue to keep in mind concerns the biological significance of all of the modifications we observed in our study (de Burbure et al. 2006). Despite their statistical significance, are the observed small changes in serum prolactin at all clinically relevant? To what extent do the variations in serum prolactin induced by the various neurotoxicants correlate with changes in brain function? Because prolactin has a large number of potential determinants, probably with different mechanisms of action, it is a rather delicate intellectual exercise to give a correct interpretation of the observed changes in terms of the possible development of neurotoxicity.

Although the lack of specificity of prolactin reduces the immediate usefulness of these dopaminergic biomarkers, the question of the potential clinical impact of the small but significant changes in terms of neurotoxicity (de Burbure et al. 2006) certainly remains an important question that further research will have to address.
